# Pathway-based approach using hierarchical components of rare variants to analyze multiple phenotypes

**DOI:** 10.1186/s12859-018-2066-9

**Published:** 2018-05-08

**Authors:** Sungyoung Lee, Yongkang Kim, Sungkyoung Choi, Heungsun Hwang, Taesung Park

**Affiliations:** 1 Interdisciplinary Program in Bioinformatics, Seoul National University, Seoul, South Korea; 2 Department of Statistics, Seoul National University, 1 Gwanak-ro Gwanak-gu, Seoul, 08826 Korea; 3 Department of Psychology, McGill University, Montreal, Canada

**Keywords:** Pathway-based analysis, Next-generation sequencing data, Multivariate analysis, Generalized structured component analysis, Hierarchical analysis

## Abstract

**Background:**

As one possible solution to the “missing heritability” problem, many methods have been proposed that apply pathway-based analyses, using rare variants that are detected by next generation sequencing technology. However, while a number of methods for pathway-based rare-variant analysis of multiple phenotypes have been proposed, no method considers a unified model that incorporate multiple pathways.

**Results:**

Simulation studies successfully demonstrated advantages of multivariate analysis, compared to univariate analysis, and comparison studies showed the proposed approach to outperform existing methods. Moreover, real data analysis of six type 2 diabetes-related traits, using large-scale whole exome sequencing data, identified significant pathways that were not found by univariate analysis. Furthermore, strong relationships between the identified pathways, and their associated metabolic disorder risk factors, were found via literature search, and one of the identified pathway, was successfully replicated by an analysis with an independent dataset.

**Conclusions:**

Herein, we present a powerful, pathway-based approach to investigate associations between multiple pathways and multiple phenotypes. By reflecting the natural hierarchy of biological behavior, and considering correlation between pathways and phenotypes, the proposed method is capable of analyzing multiple phenotypes and multiple pathways simultaneously.

## Background

In the past decade, genome-wide association studies (GWAS) have played a key role in identifying genetic associations between Single Nucleotide Variants (SNVs) and many complex biological pathologies, including type 2 diabetes (T2D), heart disease, and schizophrenia [1–3]. However, large-scale genetic analyses continue to suffer from incomplete association, of single nucleotide variants (SNVs), with distinct phenotypes (“missing heritability”), and difficulties of biological interpretation [4].

Among many proposed solutions to solve the missing heritability problem, many researchers have focused on “rare variants”. Methods for rare variants analysis arose from extending individual variant-level approaches to those at the gene-level [5, 6], and extending those at the gene level, to multiple phenotypes [7–9].

As the number of publicly available biological resources is increasing, recent methods for analyzing rare variants utilize pathway knowledge as a priori information. Since most biological behaviors manifest from a complex interaction of biological pathways [10, 11], analyzing pathway information for identifying rare variants has several advantages. In contrast to variant-level analysis, the number of statistical tests is substantially smaller in pathway analysis, resulting in less strict multiple testing corrections. Moreover, since pathways explain curated biological behaviors with multiple genes, it is easier to interpret statistically significant pathways than variant- or gene-level analyses. In this respect, many pathway-based approaches have been proposed especially using the pathway databases, which resulted in improvement of the interpretation of discoveries [12, 13].

Another effort to enhance the power of rare variants is to develop multivariate analysis methods. In general, many complex diseases arise from multiply correlated traits. For example, according to American Diabetes Association guidelines, diabetic status is diagnosed based on four traits: fasting glucose, two hours after plasma glucose, random plasma glucose, and HbA1c [14]. In that regard, simultaneous analysis of those correlated traits offer two substantial advantages over univariate analysis. First, multivariate analysis can elevate statistical power to identify additional causal biomarkers, which are not discovered by single phenotype analysis. Second, by analyzing multiple traits at once, the required number of statistical tests can be reduced, compared to those of univariate analysis. Those advantages have been well documented in past studies of large-scale sequencing datasets [15, 16].

There have now been many applications of multivariate analysis to large-scale datasets. In particular, for variant- and gene-level analysis, many multivariate methods, for common and rare variants, have been proposed [8, 15]. Despite those efforts, only a number of pathway-based multivariate analyses have been deemed feasible. Recently, three multivariate approaches, for region-level analyses, were proposed: MARV, aSPU, and MURAT. MARV [17] uses a statistical approach, reverse regression, to investigate associations between genetic regions and multiple phenotypes, by treating phenotypes as independent variables, hence enabling rapid multivariate analysis of large-scale datasets. On the other hand, aSPU [18], extends an original concept, data-adaptive sum of powered score test, to multivariate analysis, using summary statistics from single SNVs. For multivariate extension of powerful gene-based tests, MURAT (Multivariate Rare-variant Association Test) extended the original SKAT (sequence kernel association test) method to multiple phenotypes [19]. However, it might not be adequate to apply SKAT-based methods to pathway-based analysis, as we have previously demonstrated [20]. Moreover, none of the above methods are available for multivariate pathway-based association tests for rare variants with multiple pathways. Since the established pathway databases have substantial overlap among their pathways, they may ignore significant correlations between pathways, leading to misleading biological interpretations [21, 22].

In this report, we introduce a new method, “PHARAOH-multi” (**P**athway-based approach using **H**ier**A**rchical component of collapsed **RA**re variants **O**f **H**igh-throughput sequencing data), for analyzing **multi**ple phenotypes. Previously, we proposed a component-based hierarchical model for analysis of multiple pathways with a single model [20]. Here, while keeping the advantages of our previous approach, we extend it to enable analysis of multiple traits using hierarchical components of genetic variants. In addition, the proposed model can identify associations between multiple phenotypes and multiple pathways, with a single model, in the presence of subsequent genes within pathways, as a hierarchy.

## Methods

### Exome sequencing dataset for discovery study

To demonstrate the validity of the proposed method for examining large-scale datasets with multiple phenotypes, in real (biological) data analysis, we analyzed whole-exome sequencing (WES) data from a Korean population study. In brief, the dataset consists of next generation sequencing of 1087 individuals’ genomes, using the Illumina HiSeq2000 platform (Illumina, Inc., San Diego, CA), selected by the Korean Association REsource (KARE) study [23], as a part of the T2D-GENES consortium. For pathway-gene mapping, we retrieved pathway information from MSigDB [24], and mapped the genes to 217, 186 and 674 pathways extracted from the Biocarta, KEGG [25] and Reactome [26], respectively.

### Exome chip dataset for replication study

For replication of the identified pathways from the discover study, an independent cohort from Koreans, the Health Examinee shared control study (HEXA), was used. HEXA is a part of the KoGES population based cohort, initiated in 2001 [27]. In total, genotypes of 3445 individuals were acquired using the HumanExome BeadChip v1.1 (Illumina, Inc., San Diego, CA). With same quality control criteria, 24,474 rare variants were used in the analysis.

### PHARAOH-multi method

**Fig. 1:**
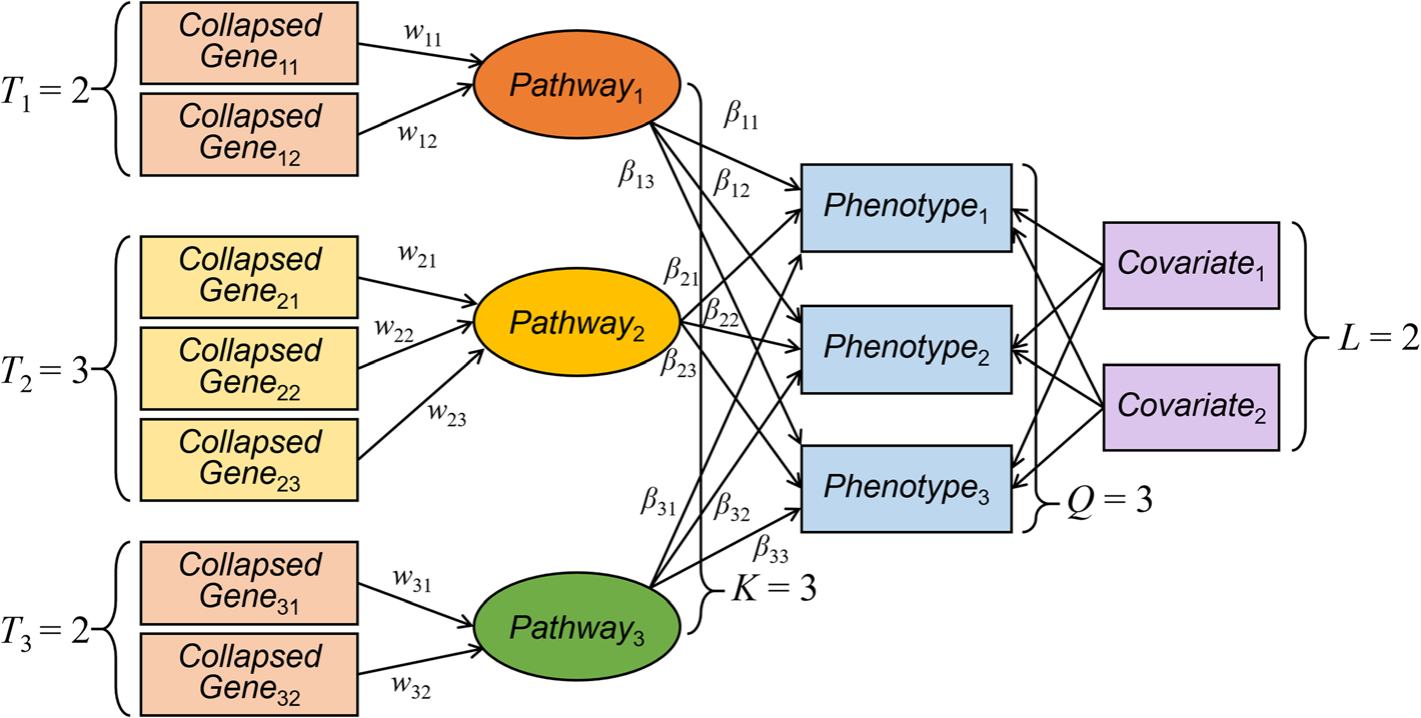
A graphical representation of PHARAOH-multi. The exemplary model is described with the number of pathways *K* = 3, the number of phenotypes *Q* = 3, the number of covariates *L* = 2, and the number of genes for each pathway *T*_1_, *T*_2_ and *T*_3_ are 2, 3 and 2, respectively. Variable *w*_*kt*_ denote the weights assigned to the collapsed genes, and *β*_*ik*_ are coefficients on the pathway latent variables. Residual terms were omitted

Our ultimate goal was to find an association between *Q* phenotypes and *K* pathways, each of whose number of genes was *T*_1_, …, *T*_*K*_, under the presence of distinct parameters for ridge penalization. The proposed method is based on Generalized Structural Component Analysis (GSCA) [28], and an exemplary structure of the model is shown in Fig. 1.

Let **Y** = [*y*_11_…*y*_1*Q*_; …; *y*_*N*1_…*y*_*NQ*_] be the matrix of phenotypes for *N* samples, where *y*_*iq*_ is the observation of the *i*^th^ sample on the *q*^th^ phenotype, and let **X** be the matrix of gene-level collapsed variables generated by summing rare variants according to their gene variant-gene mapping. Let *g*_*ij*_ ∈ {0, 1, 2} be the number of minor alleles for the *j*^th^ genetic variant of the *i*^th^ sample. Regarding the elements of X, *x*_*ikt*_ is a gene-level summary of rare variants which is defined as weighted sum of the *i*^th^ sample’s rare variants in the *t*^th^ gene of the *k*^th^ pathway, denoted by ${x}_{ikt}={\Sigma}_{j\in {M}_{kt}}{\omega}_j{g}_{ij}$, where *M*_*kt*_ is an index set that defines which rare variants are mapped onto the *t*^th^ gene in the *k*^th^ pathway. Several weighting parameters, *ω*_*j*_, can be used, as previously described in [20]. By imposing two penalty parameters *λ*_*G*_ and *λ*_*P*_ on the genes-pathway and pathways-phenotype, we sought to address potential multicollinearity problems, in both genes and pathways, in the proposed method. Such problems may adversely affect the estimation of weights and coefficients. The proposed model assumes that the phenotype, *y*_*iq*_, arises from the multivariate normal distribution with mean *μ* and covariance Σ (*i* = 1,…,*Q* and *j* = 1,…,*N*). Then we define the proposed PHARAOH-multi model as.

1 $$ {y}_{iq}={\beta}_{0q}+\sum \limits_{k=1}^K\left(\sum \limits_{t=1}^{T_k}{x}_{ik t}{w}_{tk}\right){\beta}_{kq}+{\tilde{\epsilon}}_i={\beta}_{0q}+\sum \limits_{k=1}^K{f}_{ik}{\beta}_{kq}+{\tilde{\epsilon}}_i={F}_i{\tilde{\beta}}_q+{\tilde{\epsilon}}_{i.} $$

Here, ${f}_{ik}={\sum}_{t=1}^{T_k}{x}_{ik t}{w}_{tk}$ and *F*_*i*_ indicate the *i*^th^ observation’s score of the *k*^th^ pathway, and its matrix form across *Q* phenotypes, respectively. Moreover, ${\overset{\sim }{\beta}}_q=\left[{\beta}_{0q}\ {\beta}_{1q}\cdots {\beta}_{Kq}\right]$ is a vector of coefficients for the *q*^th^ phenotype, and ${\overset{\sim }{\epsilon}}_i=\left[{\epsilon}_{i1}\cdots {\epsilon}_{iQ}\right]$ is a vector of residuals for the *i*^th^ sample.

### Parameter estimation

The proposed model seeks to associate pathways and phenotypes. The effect of the *k*^th^ pathway, on multiple phenotypes, can be determined by testing all coefficients of the pathways simultaneously (H_0_ : *β*_*k*1_ = … = *β*_*kQ*_ = 0).

Moreover, by its nature, the proposed method can further assess three more associations: (1) the effect of a gene on multiple phenotypes conditioned by a given pathway; (2) the effect of a gene on a phenotype conditioned by the pathway; and (3) the effect of a pathway on a phenotype. Detailed characteristics of the proposed model (PHARAOH-multi), including relationships and coefficients, are shown in Table 1.

**Table 1:** Parameters related to specific relationships for the proposed model

		Coefficients			Coefficients
Relationship	*P*_*k*_ *→ Y*_***∗***_	*β*_*k*1_, ..., *β*_*kQ*_	Relationship	*G*_*tk*_ *→ Y*_***∗***_	*w*_*tk*_*β*_*k*1_, ..., *w*_*tk*_*β*_*kQ*_
	*P*_*k*_ *→ Y*_*q*_	*β*_*kq*_		*G*_*tk*_ *→ Y*_*q*_	*w*_*tk*_ *β*_*kq*_

*P*_*k*_ indicates the *k*^th^ pathway, *Y*_*q*_ is the *q*^th^ phenotype, Y_*_ indicates all phenotypes, and *G*_*tk*_ indicates the *t*^th^ gene in the *k*^th^ pathway

Let *B* is a matrix of ${\overset{\sim }{\beta}}_1,\cdots, {\overset{\sim }{\beta}}_Q$. From the above model, we seek to maximize the penalized log-likelihood function, to estimate the parameters *w*_*tk*_ and *β*_*kq*_, subject to the conventional scaling constraint $\sum \limits_{i=1}^N{f}_{ik}^2=N$ [29]. The penalized log-likelihood function is expressed to

2 $$ \mathrm{\ell}\left(B\kern0.5em ,\kern0.5em W,\sum \left|{Y}_i,X\right.\right)=-\frac{NQ}{2}\log\ \pi -\frac{N}{2}\log\;\det \sum -\frac{1}{2}\sum \limits_{i=1}^N{\left({Y}_i-{B}^{\hbox{'}}{F}_i\right)}^{\hbox{'}}{\sum}^{-1}\left({Y}_i-{B}^{\hbox{'}}{F}_i\right)-\frac{1}{2}{\lambda}_G\sum \limits_{k=1}^K\sum \limits_{t=1}^{T_k}\left\Vert {w}_{tk}\right.\left\Vert {}_2\right.-\frac{1}{2}{\lambda}_G\sum \limits_{q=1}^Q\sum \limits_{k=1}^k\left\Vert {\beta}_{kq}\left\Vert {}_2\right.\right. $$

where *λ*_*G*_ and *λ*_*P*_ are the penalty parameters for each specific gene and pathway, respectively, and ‖*w*_*tk*_‖_2_ and ‖*β*_*kq*_‖_2_ are the ridge penalty function.

We previously introduced an iteratively reweighted least square (IRLS) method to minimize an univariate version of (2) under the presence of ridge penalties [20], which is similar to the alternating regularized least-squares algorithm [30]. Here we extend the previous algorithm to multivariate analysis. Let *R*_*i*_ be a “column-trimmed” matrix of GSCA [30], defined by *F*_*i*_ ⊗ *I*_*K*_, where ⊗ is Kronecker product, and *I*_*K*_ is *K* × *K* identity matrix. Maximization of (2) in respect of *B* and *W* is equivalent to minimizing the following least-square functions:

3 $$ {\displaystyle \begin{array}{l}{\phi}_B=\sum \limits_{i=1}^N{\left({Y}_i-{B}^{\hbox{'}}{F}_i\right)}^{\hbox{'}}{\sum}^{-1}\left({Y}_i-{B}^{\hbox{'}}{F}_i\right)+{\lambda}_p\sum \limits_{q=1}^Q\sum \limits_{k=1}^K\parallel {\beta}_{kq}{\parallel}_2\\ {}\kern1em =\sum \limits_{i=1}^N{\left({Y}_i-{R}_i vec(B)\right)}^{\hbox{'}}{\sum}^{-1}\left({Y}_i-{R}_i vec(B)\right)+{\lambda}_p vec{(B)}^{\hbox{'}} vec(B)\\ {}\kern1em ={\left( vec(Y)- Rvec(B)\right)}^{\hbox{'}}\left( vec(Y)- Rvec(B)\right)+{\lambda}_p vec{(B)}^{\hbox{'}} vec(B)\end{array}} $$

4 $$ {\displaystyle \begin{array}{l}{\phi}_w=\sum \limits_{i=1}^N{\left({Y}_i-{B}^{\hbox{'}}{X}_iW\right)}^{\hbox{'}}{\sum}^{-1}\left({Y}_i-{B}^{\hbox{'}}{X}_iW\right)+{\lambda}_G\sum \limits_{K=1}^K\sum \limits_{t=1}^{T_k}\parallel {w}_{tk}{\parallel}_2\\ {}\kern1em =\sum \limits_{i=1}^N{\left({Y}_i-{\Phi}_iW\right)}^{\hbox{'}}{\sum}^{-1}\left({Y}_i-{\Phi}_iW\right)+{\lambda}_G\sum \limits_{k=1}^Kw\underset{k}{\hbox{'}}{w}_k\\ {}\kern1em ={\left( vec(Y)-\Phi W\right)}^{\hbox{'}}\left( vec(Y)-\Phi W\right)+{\lambda}_G\sum \limits_{k=1}^Kw\underset{k}{\hbox{'}}{w}_k\end{array}} $$

These least-square functions are subject to diag(*R*^′^*R*) = *N*I_*NQ*_, where Φ_*i*_ is a column-trimmed matrix of *B*^′^ ⊗ *X*_*i*_ [30], and *vec*(·) is a vectorization operator. Then, it can be easily shown that the covariance matrix Σ is not related to the above equations since the PHARAOH-multi model uses multivariate linear model. An estimation of Σ can be done after convergence of *B* and *W*, by minimizing the first derivate of (2) with respect to Σ, as:

5 $$ \widehat{\Sigma}=\frac{1}{N}{\left(Y- Rvec(B)\right)}^{\hbox{'}}\left(Y- Rvec(B)\right) $$

Similarly, *B* and *W* can be updated by equating (3) and (4) to zero. This then gives the estimating equation of *B* and *W* as:

6 $$ vec\left(\widehat{B}\right)={\left({R}^{\prime }R\right)}^{-1}{R}^{\prime } vec(Y) $$

7 $$ vec\left(\widehat{w}\right)={\left({\Phi}^{\prime}\Phi \right)}^{-1}{\Phi}^{\prime } vec(Y) $$

Taken together, the overall procedure of the proposed algorithm is as follows:

1. Let *t* = 1.
2. Assign random initial values to *W*, which are then represented by *W*_(0)_.
3. Calculate *F*_(*t*)_, using *W*_(*t* − 1)_.
4. Update *B*_(*t*)_, using *F*_(*t*)_.
5. Update *W*_(*t*)_, using *F*_(*t*)_ and *B*_(*t*)_.
6. Repeat until the sum of the differences |*W*_(*t*)_ − *W*_(*t* − 1)_| + |*B*_(*t*)_ − *B*_(*t* − 1)_| converges the threshold.

Finally, we determine the values of *λ*_*G*_ and *λ*_*P*_, before applying the parameter estimation procedure. To that end, we can implement *k*-fold cross-validation (CV) to determine the values of *λ*_*G*_ and *λ*_*P*_. First, we construct a two-dimensional grid of different *λ*_*G*_ and *λ*_*P*_ values. Then we compute the deviance of each model with the given *λ*_*G*_ and *λ*_*P*_, for all CV fold values. Finally, *λ*_*G*_ and *λ*_*P*_ are selected by their average deviance, which is minimized.

### Significance testing

To assess the significance of genes or pathways, resampling methods can be used to test the statistical significance of the estimated effects of all pathways on the phenotype. In the proposed method, we utilize a permutation test to obtain *p*-values. By permuting the given phenotype, our method first generates null distributions for both pathways and gene coefficients. By computing the quantile of estimated pathway and gene coefficients, from the non-permuted dataset in each empirical null distribution, we can obtain an empirical *p*-value for any specific pathway and gene.

The testing of joint effects, between multiple phenotypes, is crucial. As shown in Table 1, PHARAOH-multi provides the individual effects of a pathway on each phenotype through *β*_*k1*_, ..., *β*_*kQ*_. The global effect of a pathway, on all phenotypes, can be evaluated by jointly testing *β*_*k1*_, ..., *β*_*kQ*_. Here, we introduce two different schema for determining a joint *p*-value for the *k*^th^ pathway, from multiple phenotypes.

Our first approach was to combine the individual *p*-values (referred as “P_K”). Since there are considerations among the estimated coefficients *β*_*k1*_, ..., *β*_*kQ*_, these correlations should be accounted for combining multiple *p*-values. Let the *p*-values from the *k*^th^ pathway be denoted by *P*_*k1*_, …, *P*_*kQ*_. The simplest way to combine those *p*-values is to use Fisher’s method, which is denoted by ${\Psi}_k=-2{\sum}_{i=1}^Q\log {P}_{ik}$ under the independence assumption. Then, the statistic, Ψ_*k*_, follows the *χ*^2^ distribution, with the degrees of freedom, 2*Q,* under the null hypothesis. An extended version of Fisher’s method, Brown’s method, can combine dependent *p*-values using a rescaled *χ*^2^distribution and covariance of *p*-values [31]. However, an analytical computation of the covariance is not feasible for large-scale datasets, due to their computational complexity. A solution for this problem [32] introduced an approximation using a third-order polynomial for the covariance, denoted by $\operatorname{cov}\left(-2\log {P}_i,-2\log {P}_j\right)\approx 3.263{\rho}_{ij}+0.71{\rho}_{ij}^2+0.027{\rho}_{ij}^3$. To that end, Kost’s approach has been shown to be one of the best working methods for combining *p*-values [33]. Here, we adopt Kost’s method by substituting *ρ* to the empirical correlation of estimated coefficients, *β*_*k1*_, ..., *β*_*kQ*_, and derive the statistic for joint effect between the *k*^th^ pathway and multiple phenotypes, as follows:

8 $$ {P}_{kost,k}=1-{\Phi}_{2{d}_k}\left({\Psi}_k/{c}_k\right), $$

where *c*_*k*_, *d*_*k*_ and ${\Phi}_{2{d}_k}$ are the scale parameter, the re-scaled degree of freedom, and the cumulative distribution function of *χ*^2^, with the degree of freedom 2*d*_*k*_ for the *k*^th^ pathway, respectively [32].

Our second approach was to construct a single statistic that combines all *Q* coefficients (referred as “P_M”). Here, we define a Wald-type statistic, ***T***, as below, and utilize ***T*** for the following permutation testing scheme:

9 $$ T=\tilde{\beta}\underset{k}{\hbox{'}}{\operatorname{cov}}^{-1}\left({\tilde{\beta}}_k\right){\tilde{\beta}}_k $$

Then, the estimated covariance $\mathit{\operatorname{cov}}\left(\widehat{{\overset{\sim }{\beta}}_k}\right)$ can be directly estimated using (6) with equation $\mathit{\operatorname{cov}}\left(\widehat{\overset{\sim }{\beta }}\right)={\left({F}^{\prime }F+{\lambda}_PI\right)}^{-1}{F}^{\prime }F{\left({F}^{\prime }F+{\lambda}_PI\right)}^{-1}\otimes \widehat{\Sigma}$ [34], or can be altered by calculating sample covariance of ${\overset{\sim }{\beta}}_k$, from permutations.

### Multiple testing correction

Since the number of pathways is far less than those of genes or genetic variants, the “multiple testing problem” remains. While Bonferroni correction can be a straightforward approach for adjusting for multiple testing, it may impose an adjustment that is too stringent, especially for correlated results [35]. To overcome this issue, we applied two types of multiple testing corrections.

First, PHARAOH-multi corrects *p*-values using the Westfall & Young permutation procedure [36], which can be easily adopted, since PHARAOH-multi already uses a permutation scheme. Let *T*_(0)_ be a vector of the statistics calculated using observed, unpermuted phenotypes, and let *T*_(*j*)_ be those from the *j*^th^ permutation. First, we rank the values of *T*_(0)_ in ascending order, and let the rank of the *k*^th^ pathway, and the *k*^th^ index, be *r*_*k*_ and *r*_(*k*)_, respectfully. Then, for each permutation *j* = 0, 1, ⋯, *J*, let ${T}_{(j)}^{\prime }$ be ${T}_{(j){r}_{(1)}},\cdots, {T}_{(j){r}_{(K)}}$, to define ${T}_{(j)}^M$ as a cumulative maximum of ${T}_{(j)}^{\prime }$. Let *I*_*j*, *k*_ be an indicator function that resolves to 1.0, if ${T}_{(0){r}_k}^{\prime }<{T}_{(j){r}_k}^M$, or 0.0, if that condition does not hold. The adjusted *p*-value for the *k*^th^ pathway, by the Westfall & Young procedure, is then defined as:

10 $$ {P}_k^{adj}=\frac{1+{\sum}_{j=1}^J{I}_{j,k}}{1+J} $$

Second, PHARAOH-multi provides False Discovery Rate (FDR) adjustment, by calculating *q*-values [37]. Here, we first obtain *K* as the number of permutation *p*-values, and from those, we can derive *q*-values, using the Benjamini-Hochberg step-up procedure.

### Simulation study

To evaluate the performance of the proposed method, we conducted simulation studies, under various scenarios. For generating rare variants, we first produced a pool of genetic variants, using SimRare [38], a rare variant simulator with well-established genetic assumptions. A pool was then generated, with default settings and gene lengths of 1Kbp. Next, we generated a simulation dataset of 10 pathways, with 1000 samples, for each replicate. All simulation scenarios were evaluated, using 1000 replicates. Based on the genotypes, the simulated phenotypes were generated by the following model, with an assumption that only the first pathway is causal to the phenotypes:

11 $$ {y}_{iq}={\beta}_{1q}{\tilde{f}}_{i1}+{\in}_{iq}={\beta}_{1q}\sum \limits_{t=1}^{H_1}{w}_{1t}{x}_{i1t}+{\in}_{iq}={\beta}_{1q}\sum \limits_{t=1}^{H_1}\left({w}_{1t}\sum \limits_{j=1}^{M_{1t}}{\gamma}_{1 tj}{g}_{i1 tj}\right)+{\in}_{iq} $$

This is then subject to diag(*F*^′^*F*) = *N*I_*K*_, where *H*_1_ is the number of causal genes in the first pathway, and *M*_1*t*_ is the number of rare variants in the *t*^th^ gene of the first pathway (i.e., causal pathway).

In the above model (eq. 11), *γ*_1*tj*_ denotes the effect of the *j*^th^ genetic variant, of the *t*^th^ gene, set to |log_10_*MAF*_*tj*_|, such that *ϵ*_*iq*_ denotes the residual and follows MVN(0, Σ). In our simulation, the settings *q* = 1,2, and *H*_1_ = 1, 2, 5, 10, were used. For each replicate, all rare variants were collapsed into genes.

## Results

For the simulation and the analysis of the real dataset, a workstation system with two Intel Xeon E5–2620 CPUs, with a combined RAM of 128GiB, were used. Note that the aSPU and MARV analyses were performed using the default settings, except that aSPU was performed without “genetic variant pruning” capability, as we observed that aSPU raises “unrecoverable error” with that capability. For our proposed method and aSPU, the number of permutations was 5000, to prevent possible lower bound limitation.

### Simulation study

For PHARAOH-multi, we selected the tuning parameters *λ*_*G*_ and *λ*_*P*_, based on three-fold CV for each replicate, using two-dimensional grids of *λ*_*G*_ and *λ*_*P*_, with six different starting points of ridge parameters, ranging from 10^1^ to 10^6^ (on a logarithmic base 10 scale). In the simulation, we considered the following conditions: number of effective genes in the causal pathway (*H*_1_), gene-level effect (*w*_*t1*_), pathway-level effect (*β*_1*q*_), and residual correlation (*ρ*). In our simulation, *H*_1_, *w*_*t1*_, *β*, and *ρ* were assumed to be 1, 2, and 5; 0.1 and 0.2; 0.1, 0.15, and 0.2; and 0, 0.25, 0.5, respectively, with evaluation of their exhaustive combinations. Other parameters, *Q*, *K* and *T*_*K*_, were fixed to 2, 10, and 10, respectively.

**Fig. 2:**
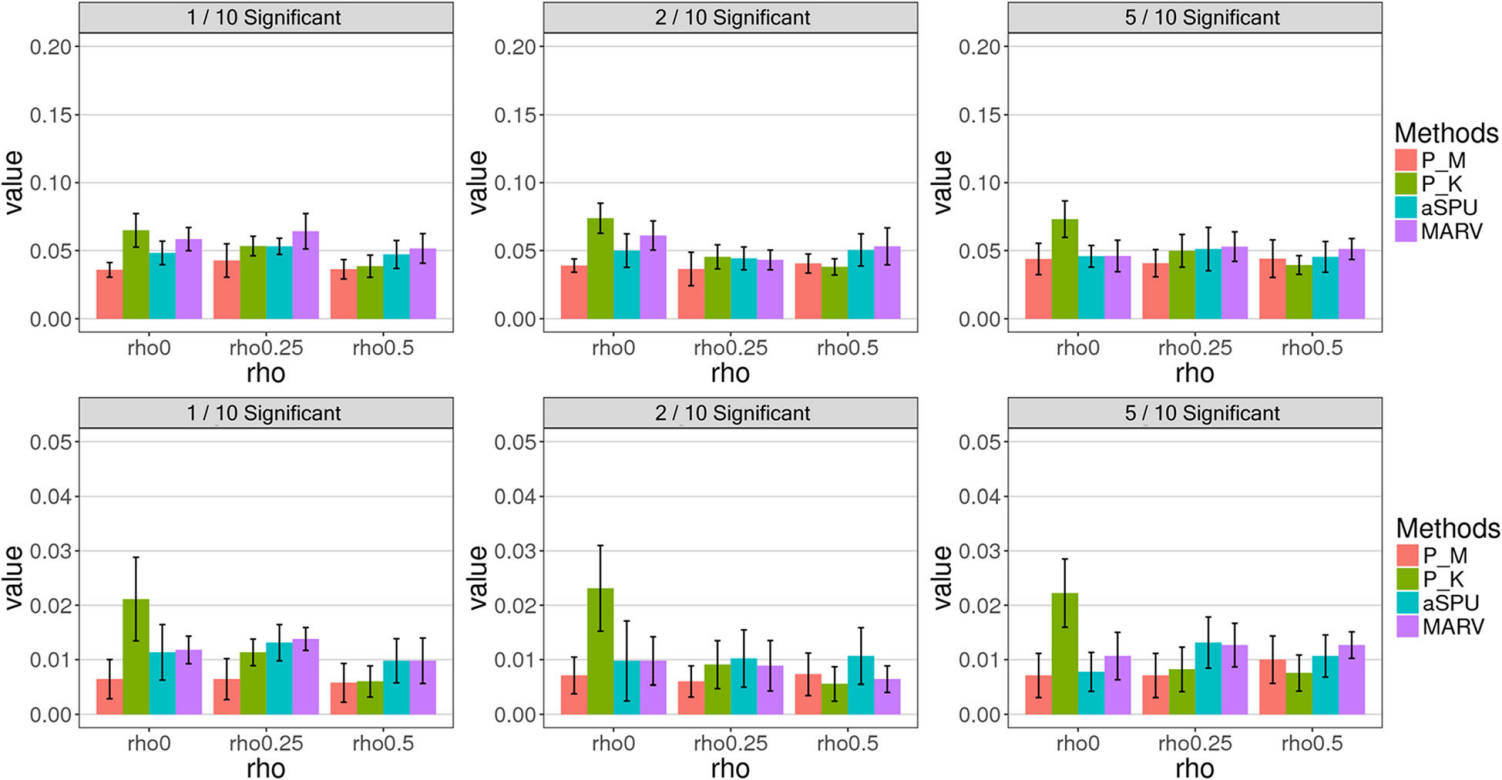
Type 1 error simulation result. Plots in the first and second row represent the type 1 error at *α* = 0.05 and *α* = 0.01, respectively. Each bar is the mean and the error bars represent SD

**Fig. 3:**
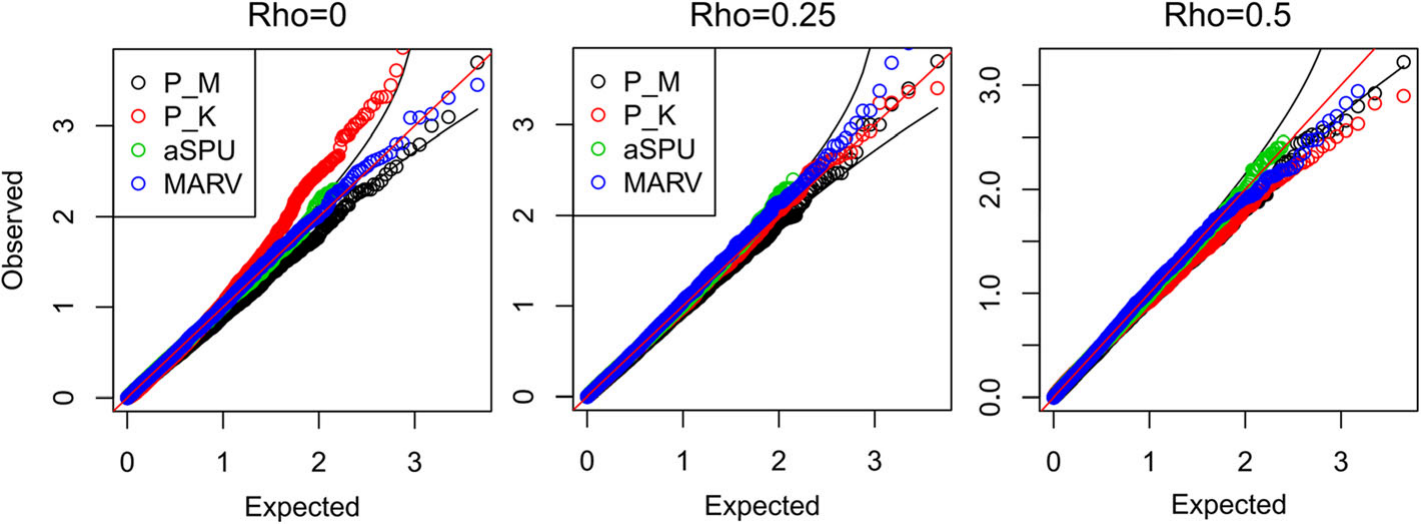
Quantile-Quantile plots of type 1 error evaluation, without multiple testing adjustment

We first compared the type 1 error rates of the proposed method vs. the traditional methods. Here, type 1 error rate was computed as a proportion of *p*-values for the pathways with no effect, and was less than the significance level, across 1000 replicates of permuted phenotypes. As shown in Fig. 2, we evaluated the type 1 errors using two significance levels, 0.01 and 0.05. As a result, type 1 errors were controlled well in the traditional methods, but PHARAOH-multi showed a moderately deflated type 1 error rate (P_M), while the inflated rate is P_K, when *ρ* = 0. In contrast, the quantile-quantile (Q-Q) plots in Fig. 3 show no inflation or deflation pattern, in all the methods, except for P_K, with no correlation between phenotypes.

It was also worthwhile to assess the gain of power in the multivariate analysis, as compared to univariate analysis. In this respect, our simulation study was conducted to compare the power gain from multivariate methods, and between multivariate and univariate analyses.

First, we checked whether PHARAOH-multi with multiple phenotypes boosts power compared to PHARAOH with a single phenotype, under the same scenarios of the power simulation. As a result, we observed that the power of PHAROH-multi was at least 2.52 times larger than PHARAOH, and this difference becomes even larger, as *w* and *β* increase (data not shown).

Second, we assessed the statistical power of PHARAOH-multi and the compared methods, defined as the proportion of the adjusted *q*-value of the simulated causal pathway (the first pathway) being less than the significance threshold, e.g., 0.05. Despite the proposed method supporting the Westfall-Young permutation procedure, it was not considered in the simulation study, due to the absence of corresponding adjustments in the compared methods.

**Fig. 4:**
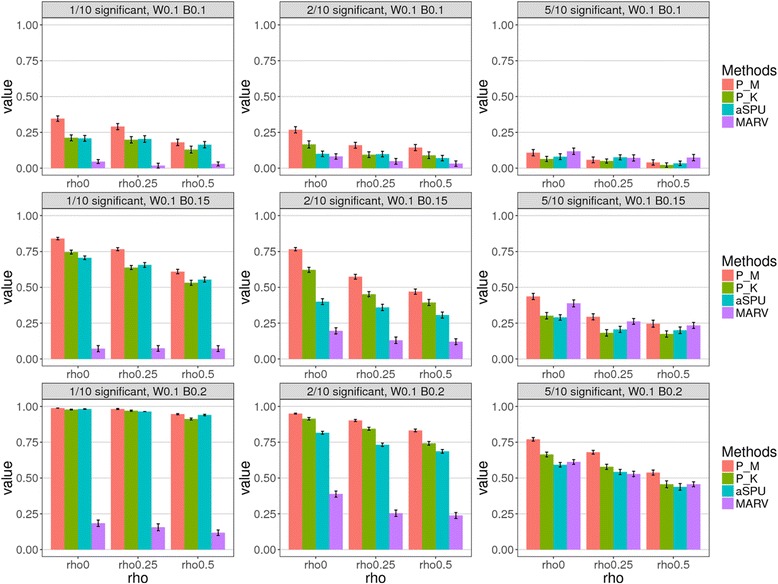
Comparison of simulation results of statistical power from various methods of multiple testing adjustment. The value of *w* = 0.1. Red and green bars represent results obtained by the PHARAOH-multi (“P_M”, using joint testing) and *p*-value aggregation (“P_K”) methods, respectively. Teal and purple bars represent the aSPU and MARV methods, respectively. Powers were calculated by the proportion of the causal pathway’s *q*-value, obtained by the Benjamini-Hochberg procedure (< 0.05). Paired *t*-test *p*-values are 7.3 × 10^− 7^ and 4.2 × 10^− 6^ for P_M vs. aSPU and P_M vs. MARV, respectively

**Fig. 5:**
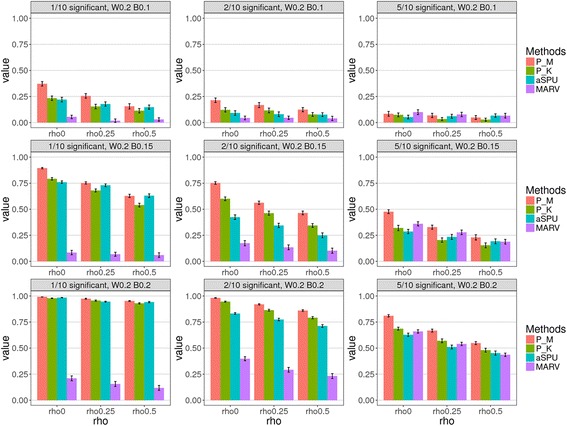
Comparison of simulation results for statistical power using multiple testing adjustment at *w* = 0.2. Red and green bars represent results obtained by the PHARAOH-multi (“P_M,” using joint testing) and *p*-value aggregation (“P_K”) methods, respectively. Teal and purple bars represent the aSPU and MARV methods, respectively. Powers were calculated by the proportion of the causal pathway’s *q*-value obtained by the Benjamini-Hochberg procedure (< 0.05). Paired *t*-test *p*-values are 3.4 × 10^− 6^ and 1.8 × 10^− 6^ for P_M vs. aSPU and P_M vs. MARV, respectively

Figures 4 and 5 show comparison results of statistical power simulation from 1000 replications. Each row in the grid of plots represents the same settings of *w* and *β*, with different numbers of causal genes in the causal pathway, and each column represents the same number of causal genes, with different effect settings.

In most scenario comparisons, the two proposed statistics obtained by PHARAOH-multi (P_M) and *p*-value aggregation (P_K) showed greater power than the other two approaches, aSPU and MARV. However, this did not hold when 50% of the genes were causal for a specific pathway, with effect sizes of *w* = 0.1 and *β* = 0.1. In order to investigate whether or not there are significant differences among powers, we performed paired *t*-tests between a pair of methods. In Fig. 4 for the case of *w* = 0.1, the *p*-values were 3 × 10^− 7^ for comparing powers of P_M and aSPU, and 4.2 × 10^− 6^ for comparing those of P_M and MARV. In Fig. 5 for the case of *w* = 0.2, the same pairwise comparison for the *p*-values were 7.3 × 10^− 7^ and 4.2 × 10^− 6^, respectively. In overall scenarios, powers of P_M were larger up to 18%p compared to aSPU in *H*_1_ = 5, *w* = 0.2 and *β* = 0.2, and were larger up to 83%p compared to MARV. Generally, P_K exhibited smaller power than P_M, and showed comparable or slightly smaller power, than aSPU.

Here, we observed three interesting patterns in the results. First, the proposed P_M and aSPU methods showed lower power, when the proportion of causal genes increase, compared to MARV. Second, the power rapidly increased, as *β* increased, as shown in Fig. 4. Third, the contribution of *w* to the power was relatively moderate, compared to *β*, as shown in corresponding scenarios of Figs. 4 and 5. The reason for the occurrence of those two patterns is that the model(s) generate phenotypes for power simulation, and eq. (11) requires the constraint of the so-called “latent variable,” in GSCA (see Methods). While both PHARAOH-multi and aSPU construct hierarchies of genes and pathways, MARV essentially treats a pathway as a large set of SNVs, since the motivation of MARV is for region- vs. pathway-based tests. The simulation setting and its overall effect on phenotypes is summarized, first at the gene-level, and then by the expression of a linear combination of those genes. In this respect, the results of PHARAOH-multi and aSPU were more plausible than those of MARV, because those two methods more properly reflected the simulation settings.

**Fig. 6:**
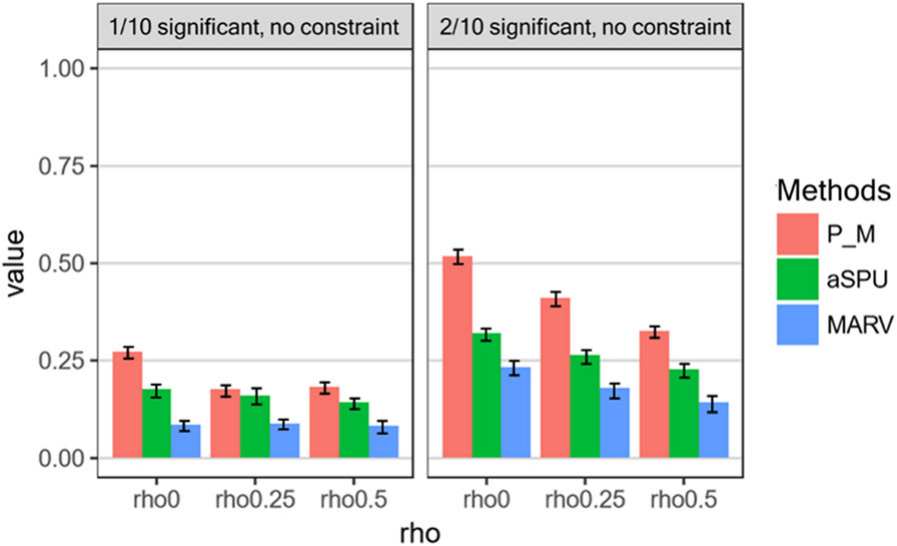
Result of additional power simulation, without constraint of manifest variables

To confirm the above hypothesis, we performed an additional comparison using the same dataset, except that the phenotypes were generated without the constraint. As shown in Fig. 6, PHARAOH-multi (“P_M”) showed larger power than in the previous simulation, while the power of MARV increased as the number of causal genes increased. However, in contrast to the previous results, the powers of PHARAOH-multi and aSPU also increased.

Finally, we investigated whether or not the statistical power changes by *M*_*kt*_. For simplicity, we split 1000 simulation datasets into two groups: the first group where the number of variants is small and the second where it is large. Then, we compared the power of each method between two groups using *t*-test. As a result, the *p*-values were 0.097 for aSPU, 0.684 for MARV, and 0.825 for PHARAOH-multi. Thus, we concluded that *M*_*kt*_ is unlikely to affect the simulation result regardless of the methods.

### Real data discovery from whole-exome sequencing dataset

To evaluate the practical performance of PHARAOH-multi, we conducted a discovery study using a large-scale sequencing dataset. Many studies suggest that the major underlying risk factors for metabolic disorders include high density lipoprotein (HDL), blood pressure (SBP, DBP), waist circumference (WAISTC), fasting glucose (FAST_GLU), and triglycerides (TG). In this regard, we conducted a multivariate analysis of metabolism-related traits, using a large-scale sequencing dataset, obtained from the Type 2 Diabetes Genetic Exploration by Next-generation sequencing in multi-Ethnic Samples (T2D-GENES) Consortium, comparing our proposed (PHARAOH-multi) and other common methods. In detail, we analyzed a dataset consisting of 1086 samples selected from the Korean Association REsource (KARE) study [23].

After removal of samples with any missing observations of the aforementioned six phenotypes, we included 1085 samples for analysis. The quality controls with genotype call rates were < 95%, or for Hardy-Weinberg Equilibrium (HWE) test *P* < 10^− 5^, the minor allele frequency was < 5% and the minor allele count was > 2, resulted in 198,761 variants. The final dataset was then mapped to genes, using the human genome-19 (hg19) reference genome coordinates, with 10Kbp flanking regions. The gene range of hg19 reference, was extracted from RefSeq track of UCSC Table Browser, as of October 2014. Finally, the gene-level collapsed variable was generated using Workbench for Integrated Superfast Association study with Related Data (WISARD), with beta-transformation weighting, as suggested in [5], with the number of genes being 4388.

**Fig. 7:**
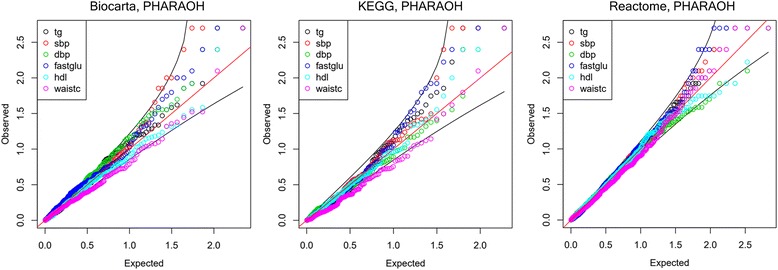
Q-Q plots of univariate PHARAOH for each pathway database

**Fig. 8:**
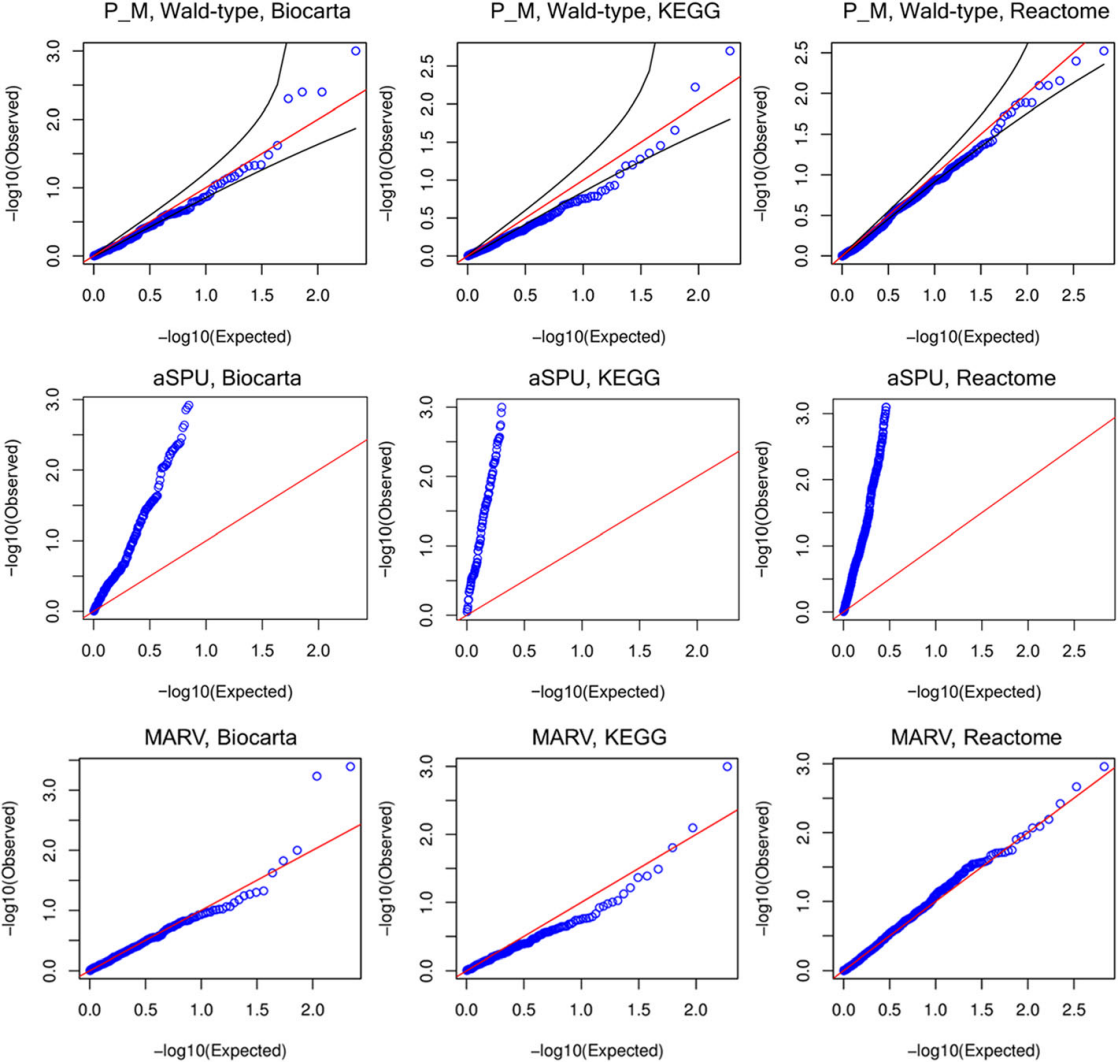
Q-Q plots of discovery study using multivariate methods. The first to third rows show the results of P_M, aSPU and MARV, respectively. The plots were drawn by unadjusted *p*-values. For each method, three pathway databases (Biocarta, KEGG and Reactome) were used. Many *p*-values of aSPU are not appear in the Q-Q plots since aSPU reports many zero *p*-value that cannot be drawn in log scale

Next, we compared our multivariate and univariate analysis results, using PHARAOH-multi and PHARAOH. As shown in Figs. 7 and 8, Q-Q plots of the results showed no substantial inflation or deflation pattern for either the multivariate or univariate results. However, with regard to pathway discovery, the results did show significant differences.

These comparisons clearly support the one advantage of multivariate analysis that we discussed above: elevation of statistical power. As with multivariate analysis, we calculated *q-*values for each univariate result. Interestingly, no univariate analysis identified significant pathways, except for SBP with KEGG, which identified three pathways (drug metabolism cytochrome P450, glutathione metabolism and progesterone-mediated oocyte maturation). As shown in Table 2, only one pathway, glutathione metabolism, was identified in the univariate analysis, and the *q-*values of univariate analyses for pathways identified by multivariate analysis, were not significant.

**Table 2:** Significant pathways of PHARAOH-multi and MARV, and their *q*-values of multivariate and univariate analyses

DB	Pathway	# of variants	Multivariate *q*-value			Univariate *q*-value (PHARAOH)					
			P_M	aSPU	MARV	tg	sbp	dbp	fastglu	hdl	waistc
KEGG											
Peroxisome		421	**0.0396**	0	0.9826	0.6886	0.7	0.9138	0.9899	0.9942	1
Glutathione metabolism		187	**0.044**	0.0076	0.9826	0.999	**0.0939**	0.9138	0.993	0.9942	1
Biocarta											
CDMAC pathway		63	**0.0858**	0.5739	0.9638	0.9817	0.1094	0.5743	0.953	0.9967	0.9962
Cell2cell pathway		112	**0.0208**	0	0.9638	0.7293	0.3063	0.5722	0.8234	0.9967	0.9962
GABA pathway		46	**0.0497**	0.0085	0.8134	0.9783	0.1094	0.5722	0.8234	0.9967	0.9962
MPR pathway		179	**0.0208**	0	0.9638	0.9783	0.1094	0.2188	0.8234	0.9845	0.9962
Caspase pathway		649	0.8358	0.1741	**0.0634**	0.997	0.8863	0.6418	0.7584	0.999	0.9928
D4GDI pathway		422	0.7626	0.3727	**0.0634**	0.997	0.9867	0.6418	0.4377	0.999	0.995
Reactome											
Glutathione conjugation		99	**0.0979**	0.0567	0.9979	0.9813	0.3859	0.9254	0.999	0.9793	0.979
Phase II conjugation		270	**0.0571**	0	0.9979	0.9813	0.3859	0.9254	0.999	0.9793	0.99

Bold numbers are the *q*-values below the significance threshold 0.1. P_M, aSPU and MARV indicate *q*-values from the joint testing method of multiple phenotypes, and univariate *q*-values indicate the *q*-values of PHARAOH analysis for each phenotype

Second, we compared the result of multivariate analyses, using PHARAOH-multi, aSPU and MARV. As shown in Fig. 8, PHARAOH-multi exhibited generally acceptable *p*-value trends, despite the result from KEGG being modestly deflated, due to the optimization of lambda. The Q-Q plots of MARV look similar to PHARAOH-multi. In contrast, aSPU showed unacceptably inflated patterns of Q-Q plots, regardless of the pathway databases, which were not used in the simulation study. This could possibly be due to substantial overlap of existing pathway databases.

As shown in Table 2, the multivariate analysis successfully identified eight pathways from three pathway databases, with Benjamini-Hochberg *q*-value < 0.1. Interestingly, PHARAOH-multi identified glutathione-related pathways in both KEGG and Reactome pathway databases, which supports the result of PHRAOH-multi. As shown in Fig. 8, the quantile-quantile plots of aSPU for the real dataset are highly inflated (i.e., their *p*-values are very small). As a result, 57.7% (Reactome), 29.5% (Biocarta) and 71.5% (KEGG) of the tested pathways by aSPU were statistically significant (*q*-value < 0.1). Unfortunately, these pathways are highly false positives. In this respect, we included the results of significant pathways identified by either PHARAOH-multi or MARV.

The identified pathways suggested evident relationships with metabolic syndrome. Since the peroxisome pathway elucidates peroxisome biogenesis, which contributes to fatty acid oxidation and biosynthesis of ether lipids, many studies have discussed interrelationship between peroxisomes and metabolic processes [39, 40]. Likewise, identification of the GABA pathway can also be explained by the relationship between GABA and peroxidation, and putative relationship of obesity [41, 42]. Moreover, identification of glutathione metabolism, and its conjugation, explain that PHARAOH-multi successfully captured a key process of metabolic disorders [43]. Finally, another report suggested a putative role of adhesion molecules in metabolic diseases, as explained by “cell2cell” pathway [44]. For the two pathways identified by MARV, we found that Caspase pathway has been known to be related to metabolic stress or perturbation [45], but no evidence for D4GDI pathway was found.

### Replication study using independent exome chip dataset

We conducted a replication study using exome chip dataset from an independent cohort, using the identified pathways in the discovery study. Despite the insufficiency of detected variants in the exome chip dataset, as a result, we successfully replicated two pathways with *p*-value < 0.1, the peroxisome pathway in KEGG (*p* = 0.059) and cell2cell pathway in Biocarta (*p* = 0.093). As shown in the literature search, the two pathways we replicated have strong relationships with metabolic disorders.

## Discussion

Compared to univariate approaches, which analyze each phenotype individually, our real data analysis successfully demonstrated that the multivariate approach could identify pathways commonly associated with specific traits. It is important to construct a systematic analysis that considers the correlation between complex diseases and their underlying biological traits. In addition, our results from two well-established pathway databases were strongly supported by many existing publications, thus demonstrating the advantage of our proposed approach.

Compared to existing multivariate analysis methods, PHARAOH-multi features several advantages. Firstly, by constructing a hierarchical structure of genes-pathways-phenotypes, four types of associations (gene-single phenotype, gene-multiple phenotypes, pathway-single phenotype, and pathway-multiple phenotypes) can be estimated simultaneously. Compared to our proposed method, existing methods of multivariate analysis were limited to gene-level analysis, and hence, the combinatorial effect of multiple genes, via biological pathways, was impossible to estimate. In addition, the proposed method considers the correlation between genes, pathways, and phenotypes, by imposing penalty parameters on the estimation procedure.

Secondly, PHARAOH-multi provides multiple options for correcting for the multiple testing issue. Although Bonferroni correction is simple, and powerfully controls type 1 error, it is a well-known fact that the Bonferroni correction often results in controls that are too stringent, when the tests are correlated. Under such conditions, application of the Westfall-Young permutation procedure can be an appropriate alternative, since its asymptotic optimality under dependence is known [35]. In this respect, the proposed method has the advantage of identifying causal pathways, by considering correlation among pathways.

For analysis times of both simulation and real datasets, MARV was the fastest among all the methods, while PHARAOH-multi ran slightly faster than aSPU. For example, in the analysis of simulation dataset of 100 genes with 1000 samples, the running times of MARV, PHARAOH-multi, and SPU were 13, 67 and 235 s, respectively. The trends of execution time were consistent regardless of simulation parameters or datasets. However, PHARAOH-multi can be further accelerated with multithreading which is not supported by MARV and aSPU. With multithreading of 8 threads, the analysis time of PHARAOH-multi was reduced to 12 s.

At this point, there are a number of subjects we can consider for future research. Our current analysis is limited only to Korean population. In our future study, we apply our method to the whole data of 13,000 WES dataset of T2D-GENES consortium [46] which contains our KARE samples. It would be a challenging work to identify novel pathways across multiple populations. For the methodological aspect, our approach uses gene-level collapsing of multiple rare variants. Although the collapsing method has the advantage that the analysis of very rare variants is possible, it cancels out the effects of variants with opposite direction (e.g, gene upregulation vs. downregulation). Despite such limitations, our method showed great potential in identifying causal genetic structure in the real data analysis. However, further research, on a more sophisticated approach that can consider the effect direction of variants, is needed. Moreover, we plan to improve our proposed multivariate analysis by applying Generalized Estimating Equations (GEE) or Linear Mixed Model (LMM). Our method can be extended to prediction models, rather than association tests, using other types of penalization, such as LASSO or SCAD [47]. Lastly, our method can also be extended to pathway interaction analysis that has been commonly performed in gene expression data analysis [48].

## Conclusion

In this study, we proposed a novel statistical approach for multivariate pathway-based analysis of rare variants, from large-scale sequencing datasets. Analyses of multiple phenotypes have been successful in analyzing various complex diseases, including type-2 diabetes (T2D) or hypertension. In general, curated guidelines suggest diagnosing T2D according to traits observed in the individual. Consequently, incorporating multiple correlated traits, to be investigated for association with specific diseases, via multivariate analysis, elevates the statistical power. In this respect, our simulation study reflects the relationship between diseases and their related traits. Throughout the simulation study, PHARAOH-multi outperformed existing multivariate methods. In addition, our proposed method successfully demonstrated several advantages of multivariate analysis, including significantly improving the detection power of causal pathways, as compared to univariate analysis, while also retaining detection power for the individual phenotype. Moreover, we successfully demonstrated that the proposed method is capable of identifying plausible pathways in the real dataset, by identifying eight pathways in the discovery study, and replicating two pathways in the replication study. We firmly believe that the proposed method will assist researchers in understanding the genetic structures that underlie many complex diseases.
